# Linked read technology for assembling large complex and polyploid genomes

**DOI:** 10.1186/s12864-018-5040-z

**Published:** 2018-09-04

**Authors:** Alina Ott, James C. Schnable, Cheng-Ting Yeh, Linjiang Wu, Chao Liu, Heng-Cheng Hu, Clifton L. Dalgard, Soumik Sarkar, Patrick S. Schnable

**Affiliations:** 1 Department of Agronomy, Iowa State University, Ames, IA 50011 USA; 2 Present address: Roche Sequencing Solutions, 500 S Rosa Road, Madison, WI 53719 USA; 3 Department of Agriculture and Horticulture, University of Nebraska-Lincoln, Lincoln, NE 68588 USA; 4 Data2Bio LLC, 2079 Roy J Carver Co-Laboratory, 1111 WOI Rd, Ames, IA 50011 USA; 5 Dryland Genetics LLC, 2073 Roy J Carver Co-Laboratory, 1111 WOI Rd, Ames, IA 50011 USA; 6 Department of Mechanical Engineering, Iowa State University, Ames, IA 50011 USA; 7 Present address: Department of Thermal Engineering, Tsinghua University, Beijing, 100084 China; 8 The American Genome Center, Uniformed Services University of the Health Sciences, Bethesda, MD 20814 USA; 9 Collaborative Health Initiative Research Program (CHIRP), Uniformed Services University School of Medicine, Uniformed Services University of the Health Sciences, Bethesda, MD 20814 USA; 10 Present address: Qiagen Sciences Inc, 6951 Executive Way, Frederick, MD 21703 USA; 11 Department of Anatomy, Physiology and Genetics, Uniformed Services University School of Medicine, Uniformed Services University of the Health Sciences, Bethesda, MD 20814 USA

**Keywords:** Genome assembly, Long molecule sequencing, Polyploid assembly

## Abstract

**Background:**

Short read DNA sequencing technologies have revolutionized genome assembly by providing high accuracy and throughput data at low cost. But it remains challenging to assemble short read data, particularly for large, complex and polyploid genomes. The linked read strategy has the potential to enhance the value of short reads for genome assembly because all reads originating from a single long molecule of DNA share a common barcode. However, the majority of studies to date that have employed linked reads were focused on human haplotype phasing and genome assembly.

**Results:**

Here we describe a de novo maize B73 genome assembly generated via linked read technology which contains ~ 172,000 scaffolds with an N50 of 89 kb that cover 50% of the genome. Based on comparisons to the B73 reference genome, 91% of linked read contigs are accurately assembled. Because it was possible to identify errors with > 76% accuracy using machine learning, it may be possible to identify and potentially correct systematic errors. Complex polyploids represent one of the last grand challenges in genome assembly. Linked read technology was able to successfully resolve the two subgenomes of the recent allopolyploid, proso millet (*Panicum miliaceum)*. Our assembly covers ~ 83% of the 1 Gb genome and consists of 30,819 scaffolds with an N50 of 912 kb.

**Conclusions:**

Our analysis provides a framework for future de novo genome assemblies using linked reads, and we suggest computational strategies that if implemented have the potential to further improve linked read assemblies, particularly for repetitive genomes.

**Electronic supplementary material:**

The online version of this article (10.1186/s12864-018-5040-z) contains supplementary material, which is available to authorized users.

## Background

The introduction of short-read DNA sequencing technologies has transformed genomic research by greatly decreasing cost while substantially increasing throughput and delivering high accuracy data. However, short-reads (100–250 bp) present challenges for the de novo assembly, haplotyping, and defining genomic structural variations [1]. These limitations are particularly problematic in genomes with high repeat content or pervasive structural rearrangements such as many crop species [2, 3].

In response to these drawbacks, long-read sequencing platforms have been developed, such as the single-molecule real-time (SMRT) sequencing approach from PacBio. Long reads can be used to span repeat-containing regions of genomes during assembly, define haplotypes and resolve structural rearrangements. However, relative to short read sequencing, SMRT sequencing suffers from high error rates (~ 15% vs. < 0.5%) [4, 5], higher costs, and more limited throughput [1]. While hybrid assembly approaches that combine short- and long-read technologies have been developed [6], combining information from multiple sequencing technologies increases cost and complexity.

New methodologies are focusing on generating synthetic long reads by taking advantage of the benefits of short-read technology but incorporating information from long strands of DNA, such as Hi-C [7] and Illumina’s synthetic long read sequencing [8]. The linked read strategy developed by 10× Genomics uses emulsion technology in conjunction with a microfluidics instrument to partition long fragments of DNA into micelles called “GEMs” (Gel Bead-In-Emulsions). Within each GEM, stretches of partitioned long DNA fragments are amplified, and a barcode unique to that GEM is added to each of these amplification products. In this way, all fragments derived from a given long DNA fragment are tagged by a shared barcode. After Illumina sequencing, the barcodes are used to identify sequences that are in close proximity in the genome. While each individual DNA fragment is typically not fully sequenced, information from many overlapping fragments derived from the same genomic region can be combined into a read cloud. Hence, long stretches of the genome can be more accurately reconstructed based on linked reads than via standard whole genome shotgun sequencing. The information retained from long fragments facilitates de novo genome assembly, haplotype phasing, and the analysis of structural variants [9].

One of the major benefits of the linked read technology is determination of haplotypes. For studies using only linked reads, the LongRanger software is available to align linked reads to human or pre-defined genomes to assist in haplotype phasing. The benefit of linked reads combined with de novo assembly is the generation of a phased de novo assembly. Thus far, a number of studies have successfully phased human haplotypes, particularly in combination with other read types [9–11]. The latest assembly algorithm from 10× Genomics, Supernova, has been used to generate diploid, phased assemblies of seven human samples using only linked reads [12]. While haplotype phasing is useful in the assembly of any species, it is likely to prove particularly valuable in the assembly of complex and/or highly heterozygous genomes. As such, it offers great promise for the assembly of plant genomes, many of which, including many major crops are polyploid [13, 14]. This is because the assembly of autopolyploid (i.e., those formed via genome doubling) and allopolyploid (i.e., those formed via the hybridization of two species) genomes requires the ability to correctly distinguish between nearly identical sequences in different regions of the same genome or between two subgenomes. Both of these challenges can potentially be overcome by linked read phasing.

While the application of linked reads technology is in its infancy, 10× Genomics has made the source code for their assembly and phasing software freely available. Multiple tools are being developed to take advantage of linked read data. For example, fragScaff [10] and ARCS [15] scaffolders have been developed to improve the many existing draft genomes by the addition of linked reads data. In addition, a simulator has been developed to generate and assess the impacts of molecule length, read number, and other linked read properties on assemblies and haplotype phasing in different genomes [16]. V_ALOR_ has been developed to assess structural variants from long range sequencing reads, including 10× Genomics, particularly for complex variants such as inversions and translocations [17].

Pervasive structural variation exists between individuals in many species [18, 19]. Detailed description of structural variants for any species requires data that are not based off a reference and has instead primarily relied upon de novo assembly. Because having additional assembled genomes prevents the biases inherent in basing genomic analyses on a single reference genome and assists in discovering variation that is not present in the current reference, efforts have focused on providing additional high-quality reference genomes, including the “1,000 Genomes Project” [20]. To date, only two non-human projects have used linked read sequencing: assembly of the 124 kb chloroplast genome of the Sitka spruce [21] and improvement of the largest conifer genome assembled to date, that of *Pinus lambertiana* [22].

Reports of de novo assembly quality in humans are confounded by the fact that most assemblies are not generated on the same cell lines or individuals. In contrast, the first report of linked read assembly in humans used two cell lines that had been sequenced by other methods [12]. The reported assembly quality for these lines was high, with an N50 of perfect matches between the linked read assembly and the corresponding assemblies of 19.8 and 16.5 kb; however, the comparison assemblies were only partial (i.e., 340 and 4 Mb, respectively). To overcome the limitation of having only partial genomes to compare, assembly error was determined by examining the inconsistencies in physical locations separated by 1 and 10 Mb of assembled de novo contig relative to the reference genome. For all assemblies, the inconsistencies were low, between 0.6 and 2%. Linked read assemblies are also able to resolve complex structural variants, as validated by mate-pair libraries [23]. These results demonstrate linked read assemblies in humans can generate relatively complete genomes with few errors. However, more work is needed to assess the use of linked reads in non-human genomes and to provide a global assessment of its assembly quality.

The maize genome serves as an excellent model to assess the quality of a genome assembly strategy such as one based on linked reads. This is because while the maize genome is more repetitive and contains more complex structural variants among individuals as compared to the human genome [3] the availability of genetically stable inbred lines makes it possible to sequence the same genome multiple time. Further, because these inbreds are fully homozygous, our analyses were focused on the accuracy of assembly in the absence of the confounding effects of heterozygosity. The maize reference genome was initially assembled using a BAC-by-BAC approach that generated a high-quality reference of the B73 inbred [3].

To assess the application of linked reads to polyploid crops we used proso millet (*Panicum miliaceum*), an orphan grain crop which was first domesticated ~ 10,000 BCE in Northern China, as a model. This cereal crop is currently grown primarily in eastern Europe, northern China, and the American west. The species is notable for its exceptionally high efficiency at converting water to grain [24]. Proso millet a tetraploid with 18 chromosomes, 2× the 9 chromosomes found in most Paniceae species and has an estimated genome size of 1 Gb [25, 26]. A recent analysis found that one of the subgenomes of proso millet likely originated from a species closely related to *P. capillare*, while the other subgenome is shared by a second *Panicum* species *P. repens* [27]. As such, proso millet is an allopolyploid. Based on limited analyses the two subgenomes are highly similar. For example, the DNA sequences of the two copies of one gene present in the both subgenomes are 94% identical [27].

Here we report the generation of de novo assemblies of the maize B73 and proso millet (var. Huntsman) genomes using 10× Genomics linked reads technology. Comparison of the B73 maize assembly to the published B73 reference genome established that linked reads can be used to assemble high accuracy contigs and scaffolds. Comparison of the proso millet assembly to the genomes of related species demonstrated that linked read technology can distinguish and separately assemble paralogous regions of this genome. These results will guide the application of linked reads technology for plant genome assembly.

## Methods

### High molecular weight DNA preparation, sequencing, and assembly

Before tissue collection one-month old greenhouse grown maize plants were transferred to a dark growth chamber for 48 h to minimize the extraction of chloroplast DNA. Maize and millet leaves were separately harvested and ground to powder in liquid nitrogen using a mortar and pestle. DNA was extracted from the resulting tissue using the Gentra Puregene Cell Kit [Qiagen (Valencia, CA), No. 158745] following the manufacturer’s protocol modified as follows to reduce the risk of shearing long DNA molecules. All mixing was accomplished via gentle inversion. Briefly, 3 mL of Cell Lysis Solution were mixed with 100 mg of ground tissue, followed by a 60-min incubation at 65 °C. RNA was removed by adding 15 uL of RNase A Solution, followed by incubation at 37 °C for 15 min. Samples were cooled to room temperature and after adding 1 mL Protein Precipitation Solution thoroughly mixed before being centrifuged at 2000×g for 10 min, after which the supernatant was decanted into a new tube containing 3 mL of 100% isopropanol. After gentle inversion, the sample was centrifuged at 2000 x g for 5 min. The isopropanol was decanted, and the remaining pellet washed with 3 mL of 70% ethanol, followed by centrifugation at 2000 x g for 5 min. After decanting the ethanol and air drying, the pellet was re-suspended in 200 uL of DNA Hydration Solution. The extracted DNA molecules were visualized via pulsed field gel electrophoresis on a CHEF-DR II [Bio-Rad (Hercules, CA)] instrument run at 6 V/cm with a 0.1- to 40-s pulse time for 16 h. Sizes were determined via comparison to the Lambda PFG Ladder (New England Biolabs (Ipswich, MA) No. N0341S) (Additional file 1: Figure S1). This gel image suggests that the DNA molecules in our input sample included not only the desired large molecules but also some that are substantially smaller.

The resulting high molecular weight DNA was prepared for GEM library creation following the standard protocol from Chromium Genome Reagent Kit User Guide Rev. A (CG00022 RevA) using the Chromium Genome Chip Kit [10× Genomics (Pleasanton, CA) No. PN-120216] and the Genome Library, Gel Bead & Multiplex V1 Kit [10× Genomics (Pleasanton, CA) No. PN-120229] with the modification of using 0.9 ng of genomic DNA input (~ 355 genome equivalents to maize). The fragment size of the prepared library was assessed using a Fragment Analyzer Automated CE System [Advanced Analytical (Ankeny, IA)] with the NGS High-Sensitivity Analysis Kit [Advanced Analytical (Ankeny, IA), Cat# DNF-486]. Each library was sequenced on a single lane of HiSeq X Ten, which generated 370,544,466 and 365,707,243,150 bp paired end reads for maize and proso millet, respectively, which were assembled into scaffolds using the Supernova Assembler version 1.1.0 for maize and the Supernova Assembler version 1.1.5 for proso millet [12], setting the “style” parameter to “pseudohap” for each. Version 1.1.0 and 1.1.5 of the Supernova Assembler generated nearly identical contigs for the maize assembly. 10× sequencing reads for both maize and proso millet were deposited into NCBI Short Read Archive (SRA) under accession SRP117789.

### Alignment to the B73 reference genome

The linked reads were aligned to the nuclear B73 reference genome AGPv2 [3] using both GSNAP, an alignment program that does not use linked read information, and LongRanger V2.1.3 (https://support.10xgenomics.com/genome-exome/software/pipelines/latest/installation), which incorporates linked read information. For the paired end GSNAP alignment, only confidently mapped reads were used for subsequent analyses. These are defined as those which were uniquely mapped with at least 50 bp aligned, at most 2 mismatches every 40 bp and tail of less than 3 bp for every 100 bp of read length.

Prior to aligning with GSNAP, the 10× barcodes were removed from each read using the LongRanger basic command. These debarcoded reads were then aligned to the AGPv2 reference genome. Only confidently mapped reads were used for subsequent analyses, which were those which uniquely aligned to the genome with at least 50 bp aligned, at most 2 mismatches every 40 bp and less than a 3 bp tail for every 100 bp of read.

To enable LongRanger alignments, the B73 AGPv2 reference genome was converted to a longranger reference using the mkref command. The linked reads were input directly into the software without pre-processing and without altering the default settings using the align command and specifying the AGPv2 reference. Genome coverage for both alignments was determined using the SAMtools [28] depth command with “-q” set to 15.

### Checking assembly quality via comparisons to the B73 reference genome

**Fig. 1:**
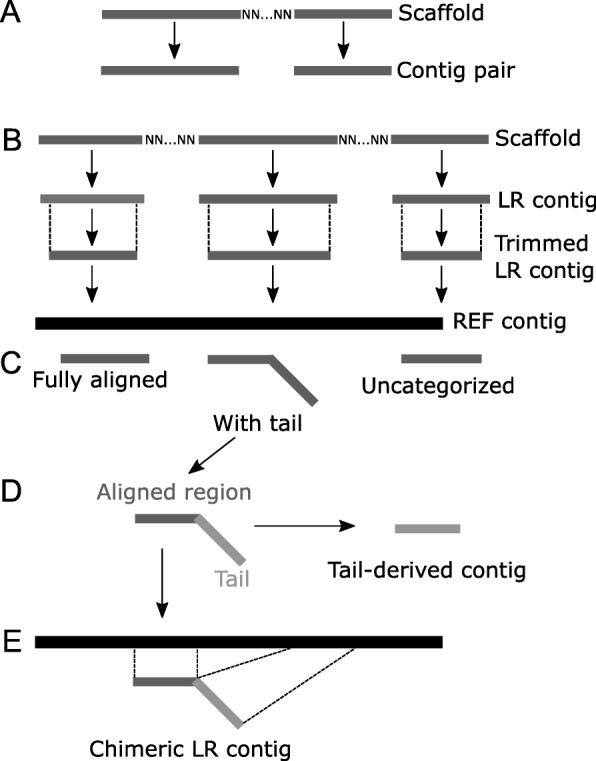
Types of assembled contigs and alignments to REF contigs. **a** A contig pair is a pair of contigs which are the only contigs originating from a single scaffold. **b** Some scaffolds contain “N”s that denote scaffolding of contigs from pairs of reads or linked reads with common barcodes. After removal of “N”s, the remaining sequences are termed LR contigs or REF contigs, depending on the origin of the scaffold. Removal of 40 bases from both ends of an LR contig results in a trimmed LR contig. **c** Trimmed or untrimmed LR contigs are aligned to the REF contigs. Alignments are categorized as fully aligned, where the entire contig aligns to a REF contig; alignments with tails, where a region of the LR contig aligns to a REF contig but a region at either or both ends of the LR contig does not align to the REF contig; or uncategorized, where the LR contig extends past the edge of a REF contig. **d** LR contigs with tails are divided into two regions: the aligned region and the tail region. Tails can be removed in silico to generate a set of tail-derived contigs. **e** LR contigs with tails that fully align to a unique location in the genome on the same or a different REF contig are termed chimeric LR contigs

Because the maize reference genome is based on the B73 inbred line, the quality of the B73 LR assembly could be assessed via comparisons to the reference genome. Assembled scaffolds contain runs of “N”s where the assembler inferred proximity without read coverage in that region. For comparison with the AGPv2 reference genome, the scaffolds were split into contigs where “N”s appeared (Fig. 1). Contigs were then aligned to the AGPv2 reference genome using BLAST with e-value set to a minimum of 1e-10 and percent identity set to a minimum of 90. The reference genome similarly contains runs of 100 “N”s that represent the junctions between REF contigs which were also split for subsequent analyses. Removing the first and last 40 bases of each sequence from the FASTA file generated trimmed LR contigs.

### Comparing contig overlaps

The GenomicRanges R package [16] was used to determine the proportion of bases covered by multiple assemblies, the overlap between contigs and MAGI v3.1 contigs [29], and the number of genes covered by the assembly. For comparisons between assemblies and with the MAGI contigs, the alignment positions to the reference genome from each set of contigs were compared to identify overlap. To determine error with the MAGI contigs, the position of the tail breakpoint for tails ≥5 bp of MAGI and LR contigs in the genome was identified. If a contig from the other dataset had an alignment that covered the tail breakpoint with no tail, this was evidence that the contig with a tail was in error (Additional file 1: Figure S6). Gene information for AGPv2 was downloaded from MaizeGDB. The start and stop positions of the canonical transcripts were used to define the location of a gene, and these positions were compared to the initial contig alignments and tail locations.

### Simulation of linked reads

The gel image of the input DNA for our empirical experiment based on the gel image (Additional file 1: Figure S1) had a modal size of ~ 50 kb, although smaller molecules were also present. Using LRSim [16] we simulated linked reads from the B73 AGPv2 reference genome that were similar and larger than our empirical data (i.e., 50 and 80 kb) and numbers of reads (400 and 800 M). As we were trying to simulate data resembling the empirical data which is from the same genotype as the reference genome, we skipped the variant simulation step by generating a fai file directly from the reference fasta file using SAMtools and starting the simulation at -u 2. All other parameters were left at their default values. Following simulation, the three sets of simulated reads were assembled and quality was assessed as described above for the empirical data.

### Machine learning

**Fig. 2:**
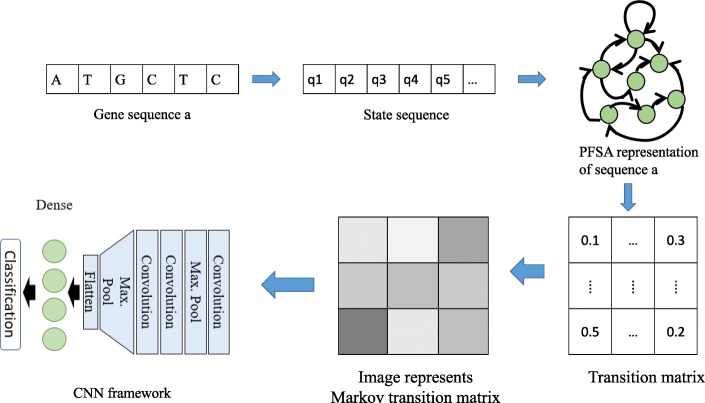
Illustration of machine learning methodology. A gene sequence is converted to a state sequence that forms a Markov chain; the Markov chain is encoded using a Probabilistic Finite State Automation (PFSA); the transition matrix of the PFSA is used as an input to the deep convolutional neutral network (CNN) for classifying the gene sequence

A hybrid machine learning Supernova framework, shown in Fig. 2, was developed based on the concepts of Markov modeling [30, 31] and Deep Learning [32] to classify the maize LR contigs. First, observable Markov transition matrices [33, 34] are learned from the gene sequences. In the context of Markov modeling, a gene sequence is inherently discrete, i.e., composed of symbols from a finite alphabet set Σ = {*A*, *T*, *G*, *C*} . Therefore, it is rather straightforward to learn a discrete Markov model from such sequences. As shown in Fig. 2, we first convert a gene sequence to a state sequence, where states are defined as a combination of D consecutive symbols. D is called the depth parameter. Therefore, if there are |Σ| (in our case, |Σ| = 4) unique symbols in a symbol sequence, we have |Σ|^*D*^number of possible states. In the results presented in this paper, we mostly use D = 1.

Upon converting the gene sequence into a state sequence, we encode the Markov chain as a probabilistic finite state automaton (PFSA). Mathematically, a PFSA is represented by a transition matrix whose elements are the transition probabilities from one state to another that can be learnt from data with a frequentist approach. The transition matrix can be treated as an image that becomes input to a deep convolutional neural network (CNN) [35, 36] in order to classify the gene sequences. In the deep CNN model, multiple convolutional layers were used with ReLU activations followed by max pooling layers and finally, a fully connected (FC) layer was used before the final output layer with ReLU activation. Dropout and batch normalization techniques were also used to resolve overfitting issues. The hyper-parameters (e.g., number of CNN layers and learning rate) for the CNN model were chosen carefully via several experiments to obtain high performance in classification.

### ABySS assembly

Linked reads contain long molecule information that Supernova uses during assembly. To determine the value of the long molecule information in assembly, LR reads were debarcoded and then assembled using ABySS Version 2.0.2, which does not make use of long molecule information. The ABySS parameters were as follows: k (size of k-mer or the span of a k-mer pair) = 85; c (minimum mean k-mer coverage of a unitig) = 10; s (minimum unitig size required for building contigs) = 200 bp; n (minimum number of pairs required for building contigs) = 10; m (minimum overlap of two unitigs) = 80 bp; p (minimum sequence identity of a bubble) = 0.95.

### Proso millet linked read analysis

Comparisons of the proso millet and foxtail millet genomes were conducted in GEvo [37] and SynMap [38] For SynMap, default parameters were used, requiring syntenic blocks to consist of at least five colinear genes with a maximum of twenty intervening gene pairs between adjacent syntenic genes. Adjacent syntenic blocks were merged using QuotaAlign merge with a maximum distance of 50 genes and filtered to keep a maximum of the two highest scoring syntenic blocks for any given foxtail millet region using the QuotaAlign algorithm with a quota setting of 2:1 proso millet:foxtail millet [39]. The “gene presence rate” was calculated as $\frac{2{G}_2+{G}_1}{2N}$, where *N* is the total number of genes under analysis (i.e., in this case 20,328), *G*_2_ is the number of observed genes with two syntenic copies in the proso millet LR assembly, and *G*_1_ is the number of observed genes with only a single assembled copy in the proso millet LR assembly. The gene missing rate is 1 - the gene presence rate. The expected gene missing rate for a single gene in a pair was calculated as 2*Ni*_*p*_ *i*_*m*_ where *i*_*m*_ is the missing rate and *i*_*p*_is the present rate. The expected number of gene pairs where both copies are identified was estimated as $N{i}_p^2$ and the expected number of gene pairs where neither copy was identified was estimated as $N{i}_m^2$. For display purposes in Fig. 4, scaffolds mapping to overlapping portions of the foxtail millet genome were tiled such that if a scaffold overlapped with a scaffold already displayed above the foxtail millet chromosome, it was placed below the foxtail millet chromosome and vice versa.

## Results

### Linked read alignment to the B73 reference genome

High molecular weight genomic DNA was extracted from maize B73 leaf tissue (Additional file 1: Figure S1) and linked read libraries were prepared using the 10× Genomics Chromium Controller and its Genome Kit V1. The Chromium linked read libraries were sequenced using one Illumina HiSeqX lane which yielded 370,544,466,150 bp paired end (PE) reads. Before assembly, we used both our standard GSNAP alignment pipeline and the 10× Genomics LongRanger alignment pipeline to align reads to the B73 reference genome to determine coverage across the genome. Based on the results from GSNAP alignment, 58.5% (433,168,634/741,088,932) of reads uniquely aligned to the B73 reference genome AGPv2 [3]. These uniquely aligned reads covered 95.9% (1,962,750,483/2,045,703,061 bases) of the reference genome. The LongRanger alignment pipeline mapped a higher percent of reads (86.2%), which is expected as this pipeline utilizes the linked read barcodes to further target alignments to defined regions of the genome. Even though LongRanger mapped 25% more reads than GSNAP, the LongRanger pipeline only increased the percent of the reference genome covered by ~ 3%, to 98.9% (2,020,251,435/2,045,703,061).

### B73 linked read assembly

Next, reads were assembled using the Supernova software. The linked read (LR) assembly contained 171,982 contigs and scaffolds ≥1 kb with an N50 of 89 kb (Additional file 1: Figure S2A). For subsequent analyses the 26,443 LR scaffolds that contained runs of “N”s denoting scaffolding were split into the corresponding LR contigs (Fig. 1). Some of the LR scaffolds were split into more than one contig; such split scaffolds/contigs were excluded from subsequent analyses. In total, after splitting scaffolds this assembly contained 234,153 contigs and had an N50 of 14.5 kb (Additional file 1: Figure S2C).

### Assessing the quality of the LR assembly

Our first approach to assess the quality of the LR assembly was to align the LR contigs to the REF contigs using NCBI BLAST v2.3.0 (Table 1). 222,531 (95.0%) of the LR contigs aligned uniquely to REF contigs with ≥95% identity. In total, 50.1% (1,038,934,839/2,045,703,061 bp) of the concatenated length of the REF contigs was covered by at least one LR contig. 25,936 contigs aligned at the end of a REF contig and hence the quality of these contigs could not be determined, and the following analyses did not consider these LR contigs. 80.8% (156,841) of the remaining LR contigs were fully aligned to REF contigs, while 20.2% (39,754 contigs) exhibited tails when aligned to REF contigs (Fig. 1). Most of these tails were, however, short relative to contig lengths (Additional file 1: Figure S2D).

**Table 1:** Alignment of contigs from different data sets to the AGPv2 reference genome using BLAST. To be classified as aligned, contigs must match the reference with ≥95% identity

No. Alignments	No. Contigs (%) from						
	LR Contigs	Trimmed LR Contigs	MAGIs	Sim 1^a^	Sim 2^b^	Sim 3^c^	ABySS
1 (Unique)	222,531 (95.0)	222,245 (95.1)	109,665 (96.1)	256,255 (98.7)	262,727 (98.8)	257,049 (98.7)	4,614,334 (42.9)
2	2145 (0.9)	2194 (0.9)	1347 (1.2)	1985 (0.8)	2014 (0.8)	1971 (0.8)	1,520,610 (14.1)
> 2	629 (0.3)	535 (0.2)	138 (0.1)	568 (0.2)	488 (0.2)	716 (0.3)	4,268,325 (39.7)
0	8848 (3.8)	8931 (3.8)	3024 (2.6)	762 (0.3)	719 (0.3)	790 (0.3)	350,313 (3.26)
Total	234,153	233,905	114,173	259,570	265,968	260,526	10,753,582

^a^Simulation 1: 50 kb molecule length and 400 M reads

^b^Simulation 2: 80 kb molecule length and 400 M reads

^c^Simulation 3: 50 kb molecule length and 800 M reads

The second approach for assessing the quality of the LR assembly was to compare the GC and repetitive content of the LR contigs and contigs from the B73 reference genome, which will be designed REF contigs (Fig. 1). To do this, we first split the scaffolded B73 reference genome into 2526 REF contigs as described above for the LR scaffolds. The LR and REF contigs had similar GC contents (46.0% and 46.9%, respectively). In contrast, the LR contigs had reduced repetitive content relative to the REF contigs (65.5% and 76.3%, respectively), as determined following a repeat masking procedure [40] even though the REF contigs are longer (Additional file 1: Table S1) suggesting that the Supernova software was less successful at assembling repetitive than non-repetitive sequences.

The third approach for assessing the quality of the LR assembly was to determine whether scaffolds were derived from single input DNA molecules. To do this we examined the relative alignment positions to the B73 reference genome of “contig pairs”, i.e., those originating from the same scaffold (Fig. 1). If an LR scaffold was correctly assembled, the contig pairs would be expected to align to the genome close to each other. Of the 7553 contig pairs, 81.4% (6147/7553) of the contig pairs that could be aligned well to the reference genome also aligned to the same chromosome. In addition, the alignment positions of 95.0% (5840/6150) of these contig pairs were within 30 kb of each other (Additional file 1: Figure S2B) suggesting that they were likely derived from the same input DNA molecule.

### Trimming LR contigs improves assembly quality

Because most of the tails associated with LR contigs are quite short (median length: 21 bp; Additional file 1: Figure S2D), we hypothesized that modestly trimming both ends of all LR contigs would substantially improve assembly accuracy. Many SNP calling pipelines employ a similar trimming strategy on individual reads to improve accuracy because the base calls at the ends of reads typically have higher error rates [41]. A potential trade-off associated with trimming is data loss. However, we estimated that ignoring 40 bases on each end of each LR contig would increase the frequency of fully aligned contigs (i.e., those without tails) from 80 to 94% with a loss of only ~ 1% of the total bases included in LR contigs (Additional file 1: Figure S3). Hence, we trimmed 40 bases from all LR contigs and aligned these trimmed LR contigs to the REF contigs. While the percentage of contigs with a unique alignment remained the same (95.0%, 222,245/233,905) (Table 1), the frequency of fully aligned contigs increased from 79 to 91% (179,237/197,049) (Table 2). 99.5% (220,150/221,364) of trimmed and untrimmed LR contigs aligned to the same genomic region. All subsequent analyses are based on trimmed LR contigs (Additional file 1: Figure S2E and F).

**Table 2:** Categorization of contig alignments

Category	No. Contigs Uniquely Aligned to REF (% of total classified)						
	LR Contigs	Trimmed LR Contigs	MAGIs	Sim 1^a^	Sim 2^b^	Sim 3^c^	ABySS
Fully aligned	156,841 (79.8)	179,237 (91.0)	101,265 (94.1)	233,396 (94.0)	240,866 (94.3)	233,758 (94.2)	4,532,012 (98.6)
With tails	39,754 (20.2)	17,812 (9.04)	6317 (5.87)	14,785 (5.96)	14,443 (5.66)	14,429 (5.81)	63,859 (1.39)
Unclassified	25,936	25,196	2083	8074	7438	8862	18,463
Total Classified	196,595	197,049	107,582	248,181	255,309	248,187	4,595,871

^a^Simulation 1: 50 kb molecule length and 400 M reads

^b^Simulation 2: 80 kb molecule length and 400 M reads

^c^Simulation 3: 50 kb molecule length and 800 M reads

### The nature of assembly errors

As discussed above, 9% of trimmed LR contigs still exhibited an alignment tail. Determining the causes for these alignment tails has the potential to provide strategies to improve future assemblies and may suggest approaches to identify contigs that are likely contain assembly errors. Alignment tails could arise via two types of assembly errors. First, the tail could be completely or partially misassembled, potentially as a consequence of repeat content. Second, the tail itself could be a correct assembly, but the junction between the tail and the initial aligned region of the contig is incorrect (i.e., a chimeric contig). To distinguish between these two possibilities, we asked whether tails could be fully aligned to other regions of the REF contigs. The tail region of each LR contig was removed in silico to create a set of “tail-derived contigs” (Fig. 1). The distribution of the lengths of the resulting tail-derived contigs is shown in Additional file 1: Figure S2F. The 11,629 tail-derived contigs longer than or equal to 30 bp were aligned to the REF contigs and categorized as described above for LR contigs. Of these, 56% (8855/11,629) aligned to the REF contigs uniquely. The percentage of tail-derived contigs that aligned uniquely to the REF contigs was lower than for LR contigs (56% vs. 95%), probably at least in part due to the short lengths of most tail-derived contigs. The percentages of uniquely aligned tail contigs (6566/8855, 74.1%) that fully aligned to the REF contigs was lower than uniquely aligned LR contigs (91%), but suggests that chimeric assembly is a major contributor to alignment tails.

The LR contigs that contain a tail-derived contig and that exhibit full alignment to unique positions in the genome will be referred to in subsequent discussions as “chimeric contigs” (Fig. 1). Of the 6566 uniquely aligned tail contigs mentioned above, 6391 were chimeric contigs (97.3%). We hypothesized that the chimeric contigs can be generated via the collapse of repeats during assembly. To test this hypothesis, we examined the repeat contents of the aligned regions of individual chimeric contigs, their tails, and the junctions between the two. Overall, the repetitive content of the fully aligned contigs was similar to that of the aligned regions and tail contigs of chimeric contigs (67.9% vs. 62.7% and 60.9%, respectively). However, the percent of junctions where the base before the breakpoint was located in a repeat region was quite high at 87.0% (5560/6391). These results suggest that chimeric contigs are formed between two correctly assembled sequences as a consequence of the assembly-induced collapse of repeats. Examining the alignments of the two regions of individual chimeric contigs show that typically both regions contain similar repeats (ex. Additional file 1: Figure S4).

If the linked read library construction strategy were successful, chimeric contigs would be expected to be assembled from adjacent regions of genome. To test this hypothesis, we examined the distance between the alignment positions of the two regions of individual chimeric contigs relative to the reference genome. For 72.2% (4741/6391) of the chimeric contigs both regions aligned to the same chromosome. Because the maize genome contains 10 chromosomes, this would be expected to happen by chance ~ 10% of the time. Hence, this result implies that the joining of two contigs into a chimeric contig is non-random. Consistent with the molecule lengths we extracted during DNA isolation, for 86.8% (4117/4741) of chimeric contigs the two regions aligned within 30 kb of each other relative to the reference genome. Hence, while the use of linked read libraries clearly enhances genome assembly, the current assembly algorithms do not fully resolve all repeat-association challenges in genome assembly.

### Using MAGIs to assess quality of LR Contigs

In the analyses reported so far, we attributed all differences between the LR contigs and the reference genome to assembly errors in the LR contigs. This approach makes the assumption that the reference genome is absolutely correct. Given the high quality of the reference genome that was generated via BAC-by-BAC sequencing this is not an unreasonable assumption, but we wanted to examine an independent assembly that would allow us to use a voting scheme to identify truth and thereby determine whether assembly errors detected via misalignment between the LR contigs and the reference genome should be assigned to the LR contigs or to the reference genome. To do this, we compared the REF contigs and LR contigs with a gene-enriched assembly (MAGIv3.1), which predates the reference genome but that has been shown to exhibit a high degree of assembly accuracy [29]. MAGI contigs are shorter than LR and REF contigs (Additional file 1: Figure S5A and B). A total of 114,173 MAGI contigs were aligned to the REF contigs as described above for the LR contigs. The MAGI and the LR contigs had similar percentages of unique alignments (96.2%, 109,665/114,173 vs. 95%, respectively) (Table 1). However, a slightly larger percentage of the MAGI contigs fully aligned to the REF contigs than did the trimmed LR contigs (94%, 101,265/107,582 vs. 91%) (Table 2).

The alignments of all 109,665 MAGI contigs and all 222,245 trimmed LR contigs that aligned to a REF contig were compared to identify assembly errors. Errors were detected as illustrated in Additional file 1: Figure S6. The region of a REF contig to which 15.6% (34,571/222,245) of the LR contigs aligned at least partially overlapped the region of that REF contig to which a MAGI also aligned. For 8.1% (2792/34,571) of LR contigs, aligned MAGI sequences provided additional evidence that the LR contig tail was a true error. MAGI contigs were more accurate than LR contigs because only 2.6% (909/34,571) of LR contigs provide evidence of MAGI sequence error. For only 0.4% (148/34,571) of LR contigs, both the LR contig and MAGI had evidence of a tail error. This could indicate both the LR contig and MAGI or the REF contigs are assembled in error. The repeat content of MAGI contigs was much lower than that of LR and REF contigs (11.6% vs. 65.5% vs 76.3%). The error as determined via alignments to MAGIs was similar to that as determined via alignment to the reference genome (92 vs. 91%).

### Gene content of the LR assembly

The quality assessments described above consider the entire assembly, but for some applications the coverage and quality of the assembly of the gene space is most relevant. We therefore asked about both the proportion of the gene space covered by the LR assembly and the accuracy of the gene space assembly. As a proxy for the gene space, we used the canonical gene transcript positions defined by the transcription start and stop sites from the filtered gene set (FGS; ZmB73_5b_FGS), a set of high-confidence maize genes. In total, 44.0% (17,440/39,656) of FGS genes were fully or partially covered by LR contigs, with 33% of the total gene space (54,190,091/161,243,349 bases) covered by LR contigs. Of those FGS genes that were fully or partially covered by LR contigs, 66.5% (11,581/17,440) were fully covered by a single LR contig, 21.1% (*N* = 3683) were partially covered by a single LR contig, and 12.5% (*N* = 2176) were covered by multiple LR contigs. Many of the FGS genes covered by multiple LR contigs (68.3%, 1731/2176) were partially covered by two or more non-overlapping contigs that were not joined by Supernova.

The existence of an LR contig tail within a gene is an indication of an assembly error. 22% (1153) of the 3683 genes partially covered by a single LR contig exhibit a tail. In 61.8% (713/1153) of these cases the assembly error is located within an intron. Thus, at least 6.8% (1153/16,995) of genes with non-overlapping contigs contain a detectable assembly error, and nearly two thirds of these errors occur in the introns which are generally more repetitive than exons. As discussed above, the accuracy of LR contigs in gene-rich regions covered by MAGI contigs is only slightly higher than the overall accuracy of the LR contigs (92% vs. 91%). The finding that 2/3 of the errors in gene-rich regions occur in repetitive introns leads us to conclude that the Supernova assembles non-repetitive regions more accurately than repetitive regions.

### Linked read coverage of contigs and the genome

As discussed above, only 58% of reads aligned uniquely to the genome, but 96% of the genome was covered by one or more uniquely aligned LR reads. In contrast, only 50% of the genome was covered by uniquely aligned LR contigs, and this percent only increased to 63% if the concatenated length of the LR contigs is considered. These results indicate either that the Supernova assembler is not incorporating some reads into contigs or that some reads are being incorrectly incorporated into contigs, or both.

Regions of the genome with no corresponding LR contig assemblies may lack sufficient read depth for proper genome assembly. To check this possibility, we compared the coverage of uniquely aligned reads to the regions of the reference genome with aligned contigs and without aligned contigs. Unsurprisingly, read coverage was higher in regions of the genome with LR contig alignment (Additional file 1: Figure S7). The depth of coverage may be the cause of whether or not a certain region of the genome is assembled. However, it is also possible that the lower coverage in regions without contigs is because these regions are repetitive with few best alignments.

Reads that align to the reference genome but not to the LR contigs are likely not being incorporated into contigs by Supernova. When examining whether reads align to the reference genome, the LR contigs, or both, 8% of aligned reads (39,081,801/519,248,347) were found to have a best alignment in the reference genome only. While these reads may not be used in LR contig assembly, they also aren’t prevalent enough to account for the ~ 45% of the genome that isn’t covered by LR contigs. An additional 30% (221,840,585/741,088,932) of all reads do not have a best alignment to either reference genome or the LR contigs and instead align to multiple locations. For these reads, it is unclear if they are being incorrectly incorporated into contigs or not used.

### Simulation of linked reads

The results presented thus far are based on a single linked read library. Certain questions are impossible to answer with only a single library, such as how often the same regions are assembled, whether increasing the length of the input DNA molecules for linked read library preparation, or whether increasing the number of reads generated from each linked read library may improve assembly. The 370 M linked reads we generated were derived from input molecules whose modal length was estimated to be > 50 kb (Additional file 1: Figure S1). Recently software (LRSim [16]) has been released to simulate linked reads. Access to simulated linked reads allowed us to evaluate if increases in the lengths of input molecules (e.g., via improvements in DNA purification) or increased depths of sequencing would affect the quality of genome assembly. Linked reads were simulated and assembled using three different sets of parameters: 50 kb molecule length with 400 M reads (Simulation 1, which was designated to match our empirical data), 80 kb molecule length with 400 M reads (Simulation 2), and 50 kb molecule length with 800 M reads (Simulation 3). The simulated reads were then assembled as described for the empirical linked reads.

The Simulation 1 and empirical assemblies had similar numbers of LR contigs with similar lengths (Additional file 1: Table S1). However, as compared to the empirical LR contigs, more of the Simulation 1 LR contigs were fully aligned to REF contigs (Table 1). In addition, fewer contigs couldn’t be assessed in the simulated LR contigs than empirical LR contigs due to aligning at the end of a REF contig.

The number of LR contigs from Simulation 1 and Simulation 2 with increased molecule lengths was similar with no apparent improvement in repeat content or molecule length (Additional file 1: Table S1). The quality of the assembled simulated LR contigs was also very similar, suggesting increasing the molecule length beyond 50 kb likely will not improve the assembly significantly. Similar to increasing the input DNA molecule length, doubling the number of sequencing reads in Simulation 3 relative to Simulation 1 did not appear to improve the amount of genome covered by the LR assembly (Additional file 1: Table S1) or the quality of the assembled contigs (Table 2). Trimming the simulation LR contigs as described for the empirical LR contigs decreased the number of simulation LR contigs with errors, but to a lesser degree (~ 2%) than observed for the empirical LR contigs (~ 10%) (Additional file 1: Table S2).

### Overlap of empirical and simulation assemblies

From the empirical data alignments, it is not possible to determine how evenly the genome was sampled by sequencing. However, the simulated data was generated evenly across the genome. If repeat content precludes assembly, the unassembled regions should be non-random and shared between simulation and empirical data experiments. The union of reference genome bases covered by aligned LR contigs from the empirical data and the three simulation experiments is 1,604,257,525. 48% of these bases are covered by LR contigs in all four data sets (Additional file 1: Figure S8A), suggesting some regions of the genome are easily assembled by linked reads. Another 18% of bases are covered by all simulation experiments but not the empirical data, suggesting there may be some genomic regions that do not have sufficient coverage for assembly in the empirical data.

In contrast, regions of the genome with errors do not appear to be shared. As the tail junction may not be identical in multiple experiments, the aligned regions of the LR contigs with tails were examined for overlap. The overlap between all experiments is low at 0.3% (201/65,383 contigs with tails). More than ½ of the LR contigs with tails from each of the experiments are unique to that experiment (Additional file 1: Figure S8B). The uniqueness of contigs with tails to an experiment only decreases by ~ 10% when the flanking 1 kb on each side of the contig alignment is also considered. This suggests that while accurately assembled regions are consistent from experiment to experiment, incorrect chimeric assembly may not occur predictably.

### Distinguishing correctly assembled contigs from chimeric contigs

Machine learning is the process of developing algorithms that “learn” the properties of large, complex data, such as identifying features of genomes [42]. A machine learning approach (Methods) was applied to the untrimmed LR contigs to determine if LR contigs with no assembly errors could be identified without alignment to a reference genome. A training set of 40,000 LR contigs, half with and half without assembly errors (as determined via alignment to the REF genome), were used as a training data set for the model. 6000 of the remaining LR contigs, half with and half without assembly errors, were tested to determine the accuracy of the model on the untrimmed LR contigs. The training accuracy and testing accuracy were 99.07% and 79.80%, respectively. 80% of fully aligned LR contigs without error were correctly classified as without error, while 71% of LR contigs with tails were correctly classified as containing errors. Accuracy of the model on the trimmed LR contigs was slightly lower (97.1% for training and 76.3% for testing), potentially due to the smaller number of contigs with tails for training and testing (32,000 instead of 40,000 for training and 2000 instead of 6000 for testing). The success of this basic implementation of machine learning in distinguishing between contigs assembled with and without errors suggests that contigs containing assembly errors may contain identifiable patterns.

### Comparison to an assembly created without long molecule information

The ABySS assembly software generated 10,753,582 unitigs, which resemble LR contigs in that they contain no scaffolding “N”s. The contigs from the ABySS assembly have a mean length of 208 bp with a median of 111 bp compared to the longer LR contigs which have a mean length of 5481 bp and a median of 2144 bp (Additional file 1: Figure S5C). ABySS does not implement a minimum length cutoff unlike Supernova. Even so, the maximum length of ABySS contigs is shorter than the LR contigs (Additional file 1: Figure S5A and C).

While it is clear that Supernova generates longer contigs using the linked read information, we wanted to confirm whether the quality or coverage of the longer Supernova contigs was compromised relative to the ABySS contigs. ABySS contigs align uniquely to the REF contigs at a much lower rate (48.4 vs 95%) than LR contigs (Table 1). This appears to be primarily a function of length, as short LR contigs are also less likely to align uniquely (Additional file 1: Figure S9). The quality of ABySS assembled contigs was higher with more contigs aligning fully than LR contigs (99 vs. 91%; Table 2). In total, the longer LR contigs covered 50.1% of the genome while the shorter ABySS contigs covered 68.7% of the genome (1,405,300,342/2,045,703,061 bases). However, the Supernova assembler does not report scaffolds less than 1 kb. When requiring a sequence length > 1 kb, the genome coverage of Supernova contigs was 49.1% for LR contigs. In contrast, only 42.9% of the genome was covered by ABySS contigs > 1 kb. In conclusion, Supernova generates longer contigs than does ABySS without sacrificing assembly quality or coverage.

### Linked reads distinguish between the two subgenomes of proso millet

Using DNA isolated from variety Huntsman, a proso millet cultivar widely grown in the western United States, a single 10× Genomics library was created and sequenced using one lane of an Illumina HiSeq X Ten instrument, resulting in 365,707,243,150 bp paired end reads. Using SuperNova Assembler a genome assembly was generated which included 30,819 scaffolds ranging in length from 1 kb to 5.6 Mb with a total length of 823 Mb (which represents 83% of the total estimated size of the proso millet genome [26]. The assembly had an L50 of 237 scaffolds and an N50 of 912 kb.

**Fig. 3:**
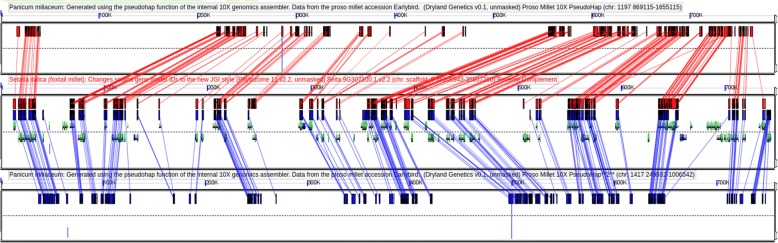
Conservation of gene order between the foxtail millet reference genome and pairs of scaffolds from the proso millet linked read assembly spanning the same region. The foxtail millet reference genome is shown in the center panel with genes indicated by gray arrows and protein coding exons by green squares. Proso millet scaffolds are shown above and below the foxtail millet genome. Red and blue lines connect gene regions from the foxtail millet genome with homologous sequence in the respective proso millet scaffolds

**Fig. 4:**
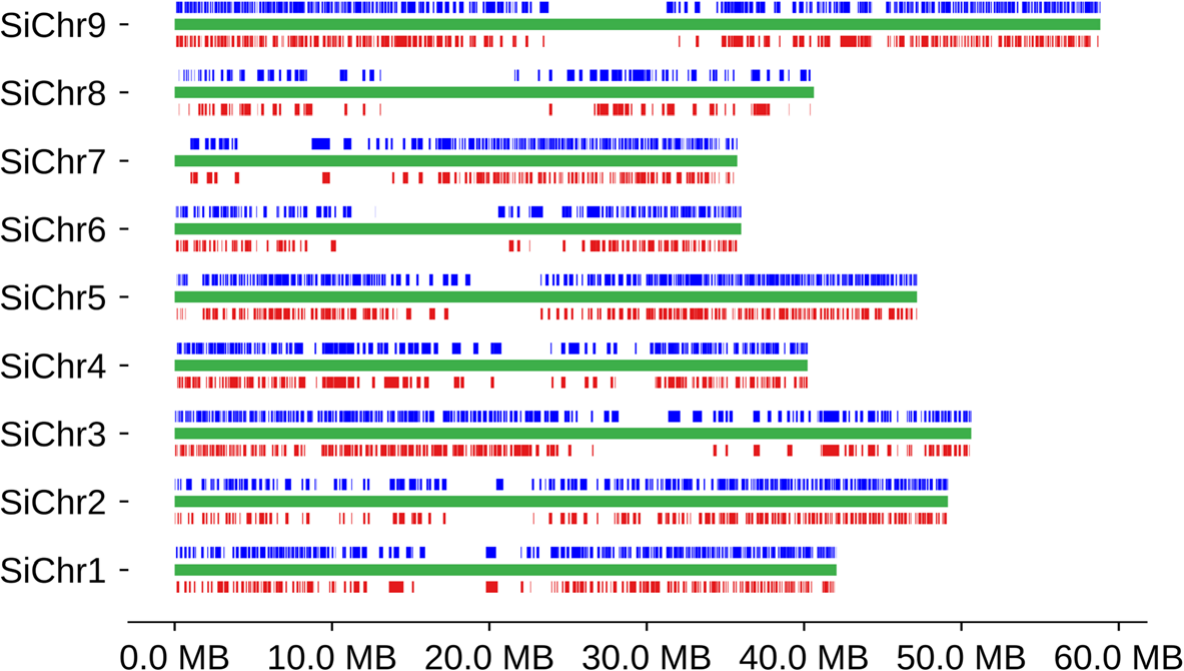
Coverage of the pseudomolecule level assembly of foxtail millet by syntenic proso millet scaffolds. Green horizontal lines indicate each of the nine foxtail millet chromosomes. Boxes in red and blue indicate syntenic regions from individual proso millet scaffolds. Boxes are tiled above (blue) and below (red) in such a way as to avoid double coverage of the foxtail millet genome by multiple scaffolds on the same side (Methods)

To assess the quality of our proso millet assembly, scaffolds were compared to the published reference genome of foxtail millet (*Setaria italica*), a close diploid relative of proso millet within the Paniceae that has nine chromosomes [43]. When two proso millet scaffolds were identified that correspond to a single region in the foxtail millet genome, gene content was well conserved both between the two proso millet subgenomes and between each subgenome and foxtail millet (Fig. 3). As shown in Fig. 4, coverage of the foxtail millet genome by syntenic regions from the proso millet assembly was highest on the chromosome arms and lowest in centromeric and pericentromeric regions. Centromeric and pericentromic regions tend to be repeat rich and gene poor. Thus, the observed pattern could result from either challenge in assembling high repeat regions, consistent with our observations of reduced repeat content and repeat-associated errors in maize, or challenges in identifying syntenic regions in gene-poor regions.

To test the effectiveness of resolving the subgenomes of proso millet, we used a set of 20,328 genes which are conserved at syntenic orthologous locations in foxtail millet and sorghum and can thus be inferred to have been present in the common ancestor of foxtail and proso millet. In 8096 cases (40%) the gene was identified as part of a syntenic block of at least five collinear genes on two separate proso millet scaffolds, indicating that for these portions of the genome both proso millet subgenomes were properly assembled. 6383 additional genes (31%) were identified as part of a syntenic block of at least five genes on only a single proso millet scaffold. These situations could have arisen when one copy of the proso millet genome was either not assembled or incorrectly assembled, or represent cases of fractionation [44], in which one of two duplicate gene copies was lost following the whole genome duplication. The remaining 5666 genes were not identified as part of any syntenic block in the proso millet assembly (25%, 5017) or the gene matched three or more syntenic regions (4%, 832).

Substantially fewer genes (expected = 9505 vs. observed = 6383; Methods) were identified in one subgenome but not the other than would be predicted by the fraction (42.1%) of conserved genes that were missing, misassembled, or placed on small contigs containing less than five genes (Methods). This indicates that the same regions of both subgenomes tend to be unassembled, misassembled, or fragmented, consistent with the observed pattern of coverage for foxtail millet chromosomes (Fig. 4, Additional file 1: Figure S10B). In contrast, maize appears to have fairly uniform coverage (Additional file 1: Figure S10A).

## Discussion

### Quality and coverage of the linked read assembly

We used linked read technology to generate a de novo assembly of the maize inbred line B73. To the best of our knowledge this study is the first published description of a de novo assembly of a non-human nuclear genome from linked reads and as such provides an opportunity to evaluate the suitability of this technology for the assembly of non-human genomes which differ substantially in structure from the human genome. Maize is an excellent model to assess the quality of an LR assembly because an independent, high-quality reference genome generated from the B73 inbred line via BAC-by-BAC sequencing is available for comparison to our LR assembly. Even though our linked reads cover > 95% of the B73 genome, the 171,982 scaffolds in our LR assembly cover only 50% of the B73 genome and, surprisingly, less (33%) of the gene space. Detailed QC of the LR contigs that comprise these scaffolds demonstrated that they were 10 times longer than contigs generated in an independent assembly of the same sequencing data but without reference to linked read information (Additional file 1: Figure S6). This length and accuracy of LR contigs and scaffolds is likely at least in part a function of Supernova’s ability to accurately exploit proximity information during assembly (Table 2, Additional file 1: Table S1). Although most (91%) LR contigs are assembled correctly, many of those errors that are present are associated with repetitive sequences, suggesting that at least some types of repeats are challenging for Supernova. Surprisingly, simulation experiments in which molecule length was increased (50 to 80 kb) or the number of sequencing reads was doubled (400 to 800 M) did not substantially improve the quality of the assembly nor the amount of genome covered (Additional file 1: Table S1). These findings also suggest that the distributions and/or characteristics of repetitive sequences in the maize genome may be limiting to the current version of Supernova. Consistent with this observation is the finding that genes without repeats are more likely to be fully covered by the assembly than are genes that contain repeats.

At least 1% of maize genes are estimated to have a nearly identical paralog (NIP) [45], and approximately half of the ~ 800 NIPs identified in the maize genome are within 200 kb of each other [3]. The 445 FGS genes covered by overlapping LR contigs may represent cases of assembly-induced collapse of NIPs and totally identical paralogs (TIPs) in the REF genome. We suspect this is because Supernova is able to exploit linked read information to distinguish closely linked copies of NIPs and TIPs that were not distinguished during the BAC-by-BAC sequencing used for the assembly of the maize reference genome.

### Comparing linked read assemblies among species

Previously the only nuclear genomes de novo assembled using LR technology have been human. Even though the human and maize genomes have similar sizes (~ 2.5 Gb vs. ~ 2.3 Gb), the human de novo LR assemblies generated scaffolds with N50 s that were 10× larger [12] than the N50 s of our maize assembly. Although the human assemblies were based on 56× coverage, as compared to our 45× coverage for the maize assembly, as discussed above, our simulation experiments suggested that coverage was not limiting N50 in maize. Instead, we believe that Supernova is challenged by the nature and/or distribution of at least some repeats present in the maize genome. The human genome has a much lower proportion of repeats with different features than the maize genome. In humans, ~ 50% of the genome is comprised of repetitive content, of which almost half are LINE elements [46]. Half of these are L1 elements, which are only ~ 6 kb when fully intact [47]. In contrast, ~ 75–85% of the maize genome is repetitive with ~ 70% of these repeats originating from ~ 1-2 kb *Copia* and *Gypsy* LTR retrotransposons [3, 48]. While the shorter length of individual maize LTR retrotransposons would be expected to be easier to assemble, these repetitive elements are often found in blocks of 20-200 kb long and form complex structures of nested repeats [49]. These blocks of repetitive content may not be completely spanned by high molecular weight DNA, and even when they are, within the blocks the same repeat may be present multiple times, making resolution of individual repeats more difficult. Furthermore, the non-repetitive sequences may be assembled but interspersed with repeats such that the assembled contigs do not pass Supernova’s minimum length requirement (1 kb). Supporting this hypothesis, the mean distance between masked repeats in the REF contigs is 431 bp, and the median is only 92 bp. In addition, only 68% of maize transcripts are longer than 1.5 kb and this may explain the reduced coverage of genes. Based on the extensive repetitive content of maize, the high coverage of the genome by aligned linked reads may seem surprising. However, while the complex repeat structure of maize makes assembly challenging, the same structures make unique alignments of individual reads more likely to be identified.

The proso millet linked read assembly covered more of the proso millet genome (83%) as compared to the maize linked read assembly (50%). The proso millet was sequenced to greater read depth (~ 107×) than maize (45×); however, doubling the number of maize reads in our simulation experiment did not increase maize genome coverage. While repeat content has not been assessed in proso millet, its much smaller genome (1 Gb) with a similar gene number to that of maize (~ 40,000) suggests that proso millet has a lower repeat content than maize and is therefore more similar to the human genome in this respect. The difference in coverage obtained between the two crops genomes is consistent with the hypothesis that repeat content limits coverage in Supernova linked read assemblies.

### Strategies to adapt LR technology for assembling complex plant genomes

Machine learning identifies patterns in complex data sets [42]. A machine learning strategy was able to distinguish with ~ 79% accuracy between LR contigs assembled with and without errors. The success of the machine learning approach to identifying contigs with errors suggests there are patterns associated with the assembly errors. Our analyses demonstrate that the quality of LR data is high and suggest that repetitive sequences are a major factor limiting accuracy and coverage in the current assembly approach. If so, LR assemblies could potentially be further improved via computational adjustments to Supernova. Supernova primarily uses LR barcode information for initial filtering of linked reads and to extend scaffolds after initial assembly [12]. For example, contig coverage and quality could potentially be improved if barcode information were more fully utilized during assembly. For instance, barcode information could be used to identify regions of scaffolds that align to more than the expected numbers of barcoded input DNA molecules, potentially as a consequence of repeat collapse; these regions could be tagged as potential errors and/or flagged for additional processing. Additionally, higher stringency during de Brujin graph construction or bubble formation steps of the Supernova assembly that use individual linked reads also has the potential to improve the assembly. Improvements to the assembly of repeats may also improve the coverage of the assembly. Our data suggest that repeat collapse is causing regions between pairs of closely linked repeats to remain unassembled (Additional file 1: Figure S4). This could result in either of two negative consequences. First, reads between the pairs of repeats may not be assembled or if the reads between the repeats are assembled the resulting contig may be shorter than the 1 kb cut-off employed by Supernova. We suspect either or both of these consequences may account for reduced coverage of the maize genome in the LR assembly and in particular the underrepresentation of short genes in the LR assembly. If so, developing strategies to avoid repeat collapse may be applied. For example, methods to assess coverage could be used to identify and correct potential cases of repeat collapse. The combination of increased use of barcode proximity information, increased stringency during assembly, and incorporating depth of read coverage to assess the accuracy of assembled contigs has the potential improve LR assembly.

## Conclusions

A large proportion of all plant species are polyploids and polyploid species have been disproportionately targeted for domestication [50]. With modern sequencing technology providing the ability to sequence and assemble genomes as large as 20 Gb [51], complex polyploids represent one of the last grand challenges in genome assembly. Here we have demonstrated that linked read technology is capable of successfully distinguishing the two subgenomes of a recent allopolyploid, thereby producing an assembly in which the two subgenomes have been correctly resolved. As expected, the two sets of paralogous scaffolds exhibit a high degree of collinearity and conservation with diploid outgroups. With an N50 of nearly one Mb, this assembly is of sufficiently high contiguity that conventional approaches such as physical or genetic maps [52], as well as other supplementary wet lab methodologies would likely produce a pseudomolecule level assembly in which the subgenomes are fully resolved.

Our assemblies of both maize and proso millet were derived from homozygous inbred lines. It should be noted however that the Supernova software is capable of handling heterozygous individuals as evidenced by the successful application of the linked read strategy to heterozygous human genomes.

## Additional file

Additional file 1:**Figure S1.** Pulsed field gel electrophoresis image of B73 high molecular weight DNA after extraction and before LR library preparation. **Figure S2.** Length distributions of A) LR scaffolds (*N* = 171,932), B) distances between contig pairs (two contigs that comprise a scaffold) which align to the same chromosome (*N* = 6566), C) LR contigs (*N* = 234,153), D) LR contig tails (*N* = 64,704), E) trimmed LR contigs (*N* = 233,095), and F) trimmed LR contig tails (*N* = 39,237). **Figure S3.** Estimated percent reduction in assembly error (A) and percent bases remaining following contig trimming (B). **Figure S4.** Repeat content at the junction of the aligned portion of a representative LR contig with a tail and its tail. **Figure S5.** Length of A) trimmed LR (N = 234,153), B) MAGI (*N* = 114,173), and C) ABySS contigs (*N* = 10,787,574). Means ($\overline{x}$) and medians ($\overset{\sim }{X}$) are indicated by vertical lines with the values reported on each plot. **Figure S6.** Comparisons of LR, MAGI, and REF contigs. MAGI and LR contigs were aligned to REF contigs. **Figure S7.** Coverage of debarcoded reads uniquely aligned to the reference genome in regions where LR contigs align (*N* = 244,649) or do not align (N = 1,324,967). **Figure S8.** Genomic overlap of LR assemblies. A) Percent of bases shared between aligned regions of fully aligned contigs from all LR assemblies. **Figure S9.** Relationship between contig length in bins of 1 kb and contig quality for LR and ABySS assemblies. **Figure S10.** Genome-wide distribution of maize and proso millet LR contigs. **Table S1.** Summary of assemblies. **Table S2.** Categorization of trimmed simulation contig alignment. (DOCX 789 kb)

## Abbreviations

CNN: Convolutional neural network
FC: A fully connected
FGS: Filtered gene set
GEMs: Gel Bead-In-Emulsions
LR: Linked read
NIPs: Nearly identical paralogs
PE: Paired end
SMRT: Single-molecule real-time
TIPs: Totally identical paralogs
