# Supplementary material for: Linked read technology for assembling large complex and polyploid genomes
Source: BMC Genomics. 2018 Sep 4;19:651. doi: 10.1186/s12864-018-5040-z (PMC6122573; doi:10.1186/s12864-018-5040-z)
Supplement: Supplementary file 1 — Figure S1. Pulsed field gel electrophoresis image of B73 high molecular weight DNA after extraction and before LR library preparation. Figure S2. Length distributions of A) LR scaffolds (N = 171,932), B) distances between contig pairs (two contigs that comprise a scaffold) which align to the same chromosome (N = 6566), C) LR contigs (N = 234,153), D) LR contig tails (N = 64,704), E) trimmed LR contigs (N = 233,095), and F) trimmed LR contig tails (N = 39,237). Figure S3. Estimated percent reduction in assembly error (A) and percent bases remaining following contig trimming (B). Figure S4. Repeat content at the junction of the aligned portion of a representative LR contig with a tail and its tail. Figure S5. Length of A) trimmed LR (N = 234,153), B) MAGI (N = 114,173), and C) ABySS contigs (N = 10,787,574). Means ($\overline{x}$x¯) and medians ($\overset{\sim }{X}$X~) are indicated by vertical lines with the values reported on each plot. Figure S6. Comparisons of LR, MAGI, and REF contigs. MAGI and LR contigs were aligned to REF contigs. Figure S7. Coverage of debarcoded reads uniquely aligned to the reference genome in regions where LR contigs align (N = 244,649) or do not align (N = 1,324,967). Figure S8. Genomic overlap of LR assemblies. A) Percent of bases shared between aligned regions of fully aligned contigs from all LR assemblies. Figure S9. Relationship between contig length in bins of 1 kb and contig quality for LR and ABySS assemblies. Figure S10. Genome-wide distribution o [file 12864_2018_5040_MOESM1_ESM.docx]

**Supplemental Figures and Tables**


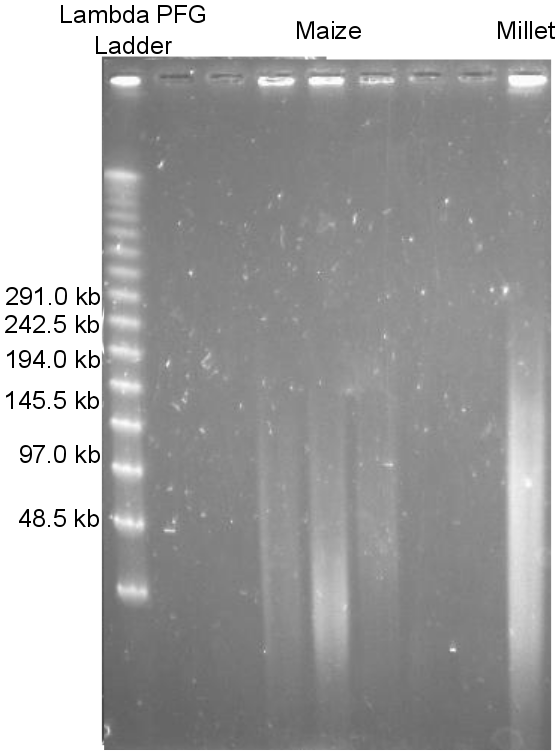


Figure S1. Pulsed field gel electrophoresis image of B73 high molecular weight DNA after extraction and before LR library preparation.

**
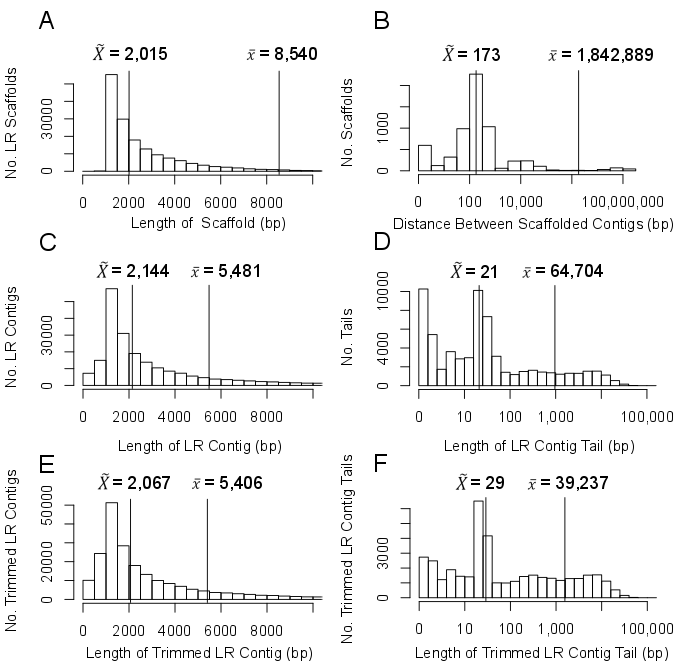
**

Figure S2. Length distributions of A) LR scaffolds (N=171,932), B) distances between contig pairs (two contigs that comprise a scaffold) which align to the same chromosome (N=6,566), C) LR contigs (N=234,153), D) LR contig tails (N=64,704), E) trimmed LR contigs (N=233,095), and F) trimmed LR contig tails (N=39,237). Means ($\bar{x}$) and medians ($\tilde{X}$) are indicated by vertical lines with the values reported on each plot. Figures A, C, and E truncate at 10 kb: 13,043 LR scaffolds, 31,812 LR contigs, and 31,591 trimmed LR contigs are greater than this size. Note the x-axis for plots B, D, and F are on a log scale.


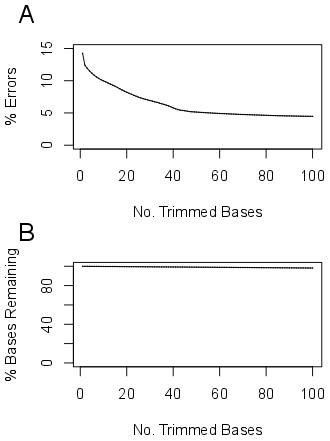


Figure S3. Estimated percent reduction in assembly error (A) and percent bases remaining following contig trimming (B). These estimates were obtained by comparing the lengths of LR contig tails with the amount of trimming required to remove the tail.


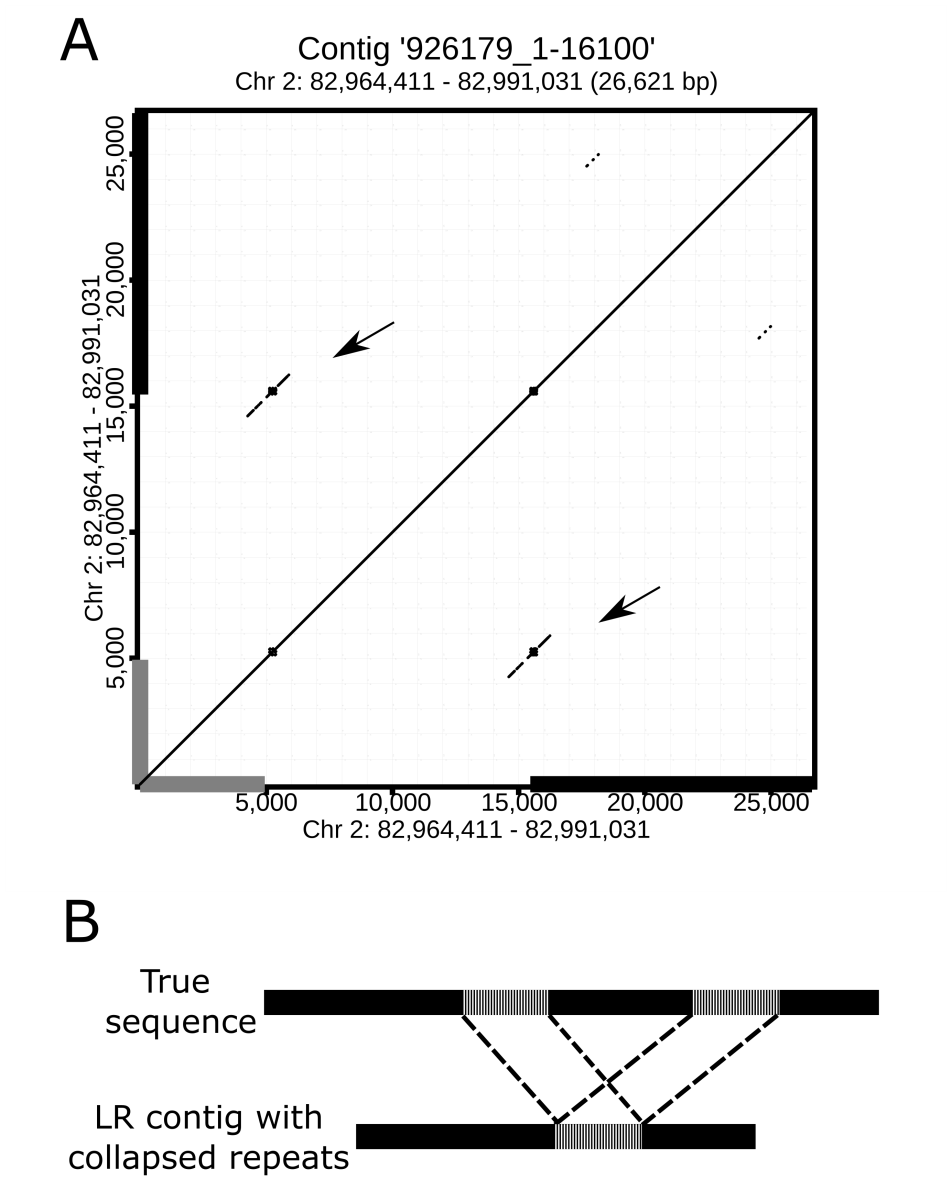


Figure S4. Repeat content at the junction of the aligned portion of a representative LR contig with a tail and its tail. A) Gray regions on the x and y axis of the dot plot represent the position of the aligned region of the contig. Black regions on the x and y axis represent the positions at which the tail aligns. Each dot represents a 50 bp window with 100% identity. Steps between windows are 1 bp. The diagonal lines of dots indicate regions where the sequences on the x and y axis exhibit high similarity. Arrows indicate duplicated regions at the ends of the contig and tail. B) True sequences that contain two similar repeats (vertical dashed region) in close proximity such as the example presented in A appear to be collapsed into a single repeat in the LR contigs.


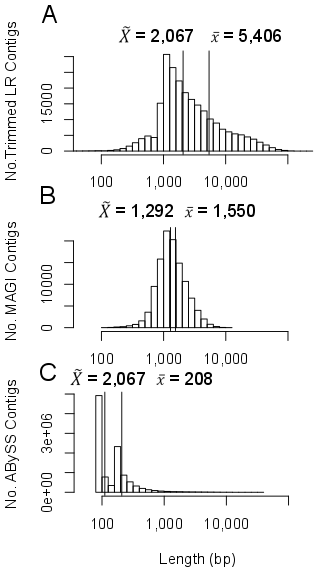


Figure S5. Length of A) trimmed LR (N=234,153), B) MAGI (N=114,173), and C) ABySS contigs (N=10,787,574). Means ($\bar{x}$) and medians ($\tilde{X}$) are indicated by vertical lines with the values reported on each plot.


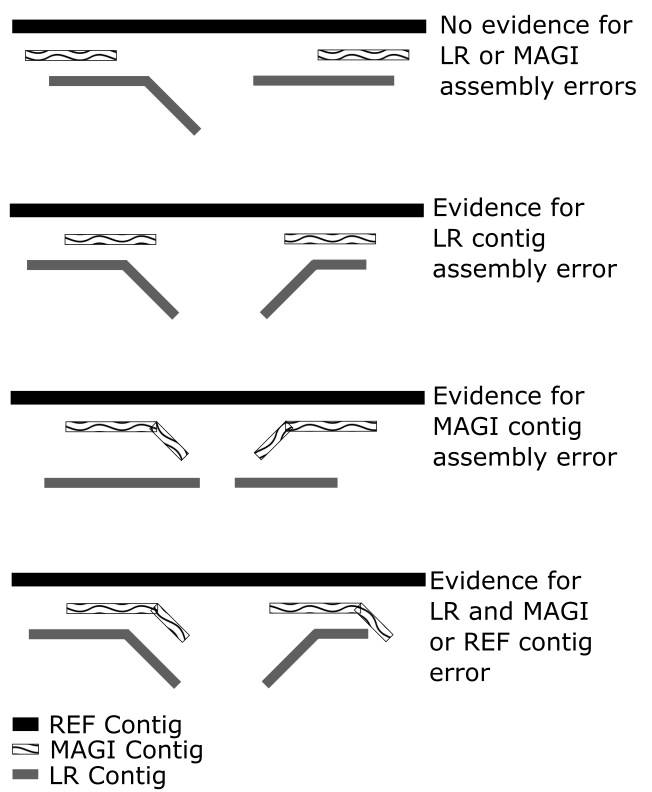


Figure S6. Comparisons of LR, MAGI, and REF contigs. MAGI and LR contigs were aligned to REF contigs.


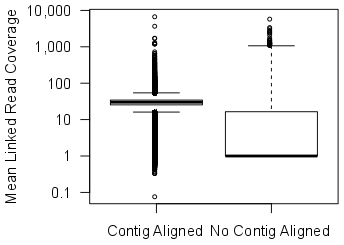


Figure S7. Coverage of debarcoded reads uniquely aligned to the reference genome in regions where LR contigs align (N=244,649) or do not align (N=1,324,967). Regions with LR contig alignment are defined by positions where the LR contigs align. While a contig may align to this whole region, not all bases have linked read coverage; hence, the average coverage can be < 1. Regions with no LR contig coverage are defined by continuous linked read coverage where no contigs align and the minimum coverage in these regions is 1.


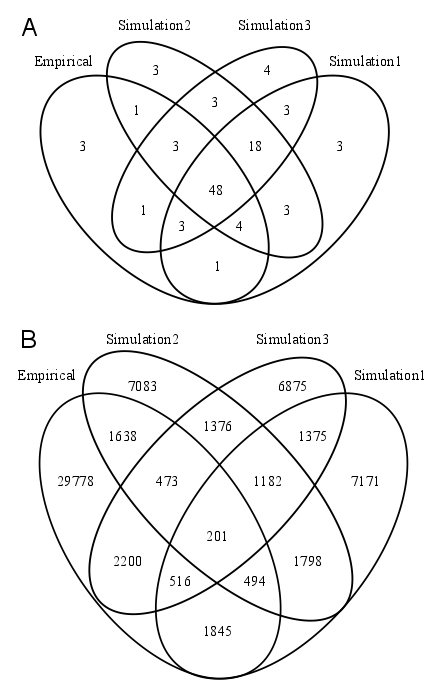


Figure S8. Genomic overlap of LR assemblies. A) Percent of bases shared between aligned regions of fully aligned contigs from all LR assemblies. The union number of genomic bases covered by at least one contig for at least one assembly is 1,604,257,525. B) Overlap of aligned regions of LR contigs with tails. Contigs are considered to be overlapping if > 1 aligned base is also aligned from another contig with a tail.


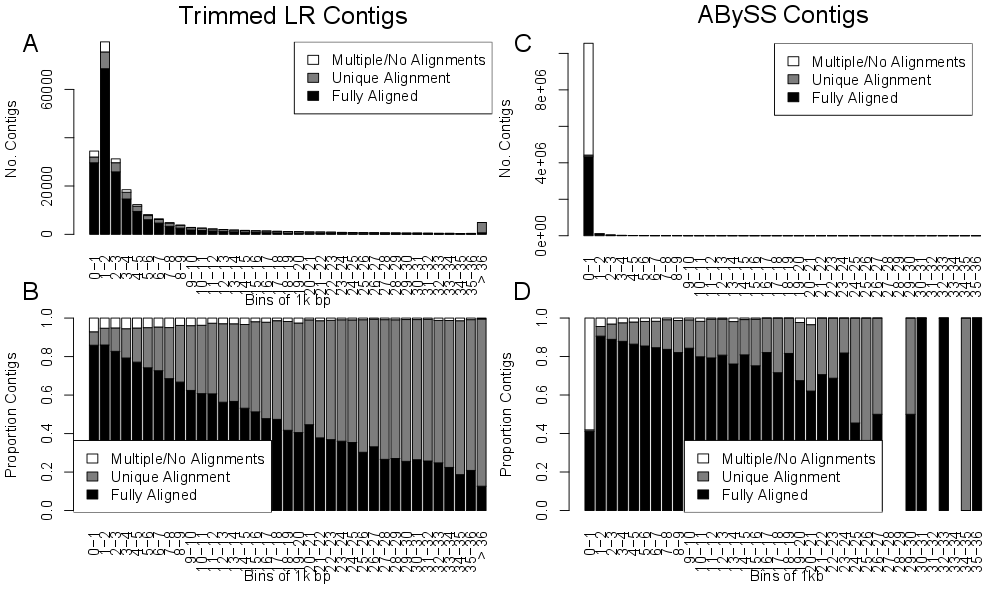


Figure S9. Relationship between contig length in bins of 1 kb and contig quality for LR and ABySS assemblies. A and B) LR contigs are more likely to have a unique alignment but not be fully aligned. C and D) Short ABySS contigs are more likely to have multiple or no alignments.


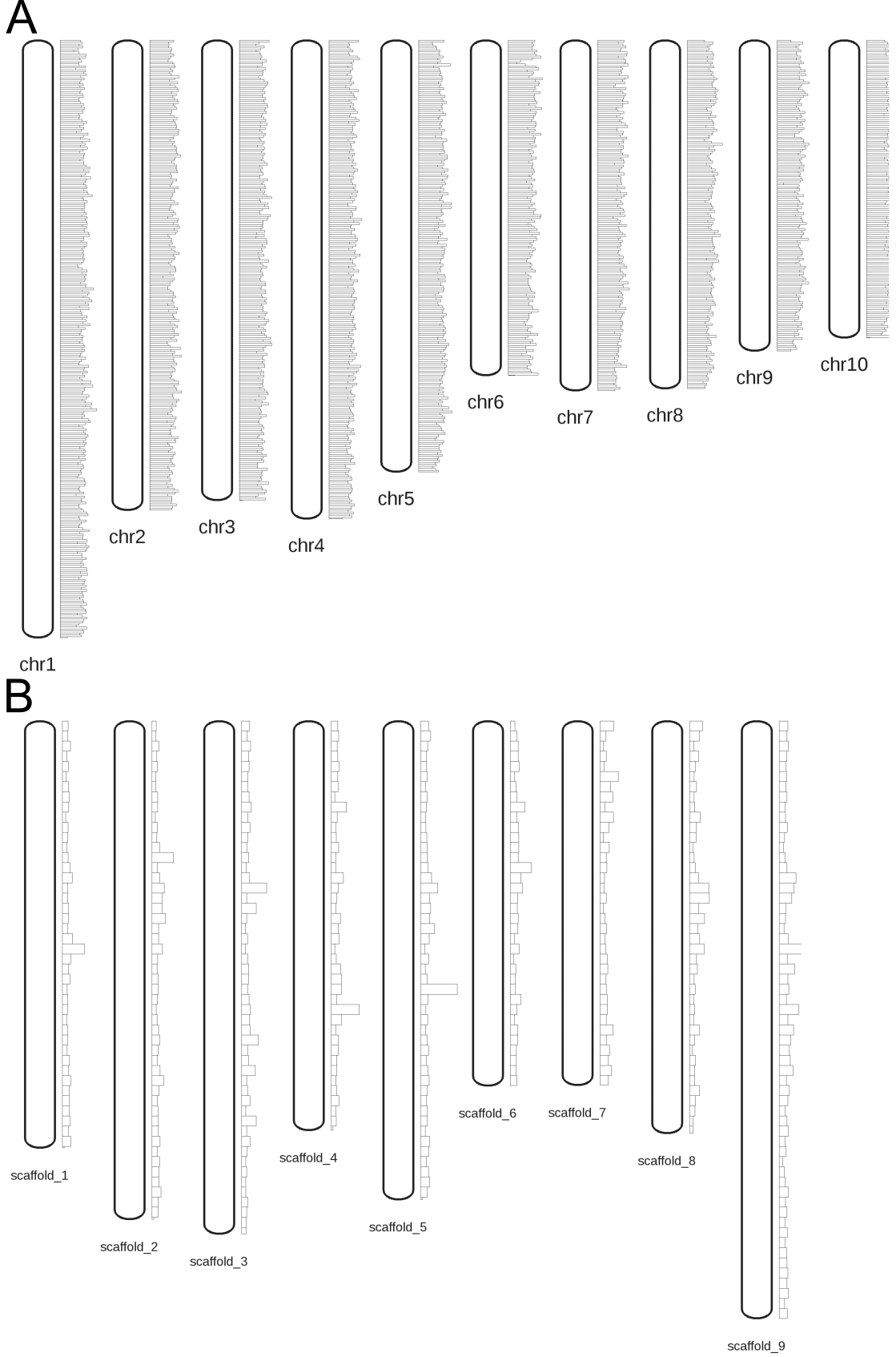


Figure S10. Genome-wide distribution of maize and proso millet LR contigs. Vertical bars to the right of each chromosome indicate the number of contigs aligned in each bin. A. Distribution of empirical B73 10X assembled contigs by 1Mb bins in B73_reference genome with ≥95% identity and ≤5% tails. The number of contigs per bin ranges from 3 to 167 with a mean of 106 and a median of 107. B. Distribution of empirical proso millet 10X assembled contigs aligned to 1Mb bins of the Setaria genome with ≥85% identity. The number of contigs per bin ranges from 0 to 381 with a mean of 78 and a median of 69.

Table S1. Summary of assemblies.

| **Data Set** | **REF^^[[1]](#footnote-1)^^** | **Empirical** | **MAGI** | **Sim 1^^[[2]](#footnote-2)^^** | **Sim 2^^[[3]](#footnote-3)^^** | **Sim 3^^[[4]](#footnote-4)^^** | **ABySS** |
| --- | --- | --- | --- | --- | --- | --- | --- |
| **No. Scaffolds** | 10 | 171,982 | NA | 200,421 | 208,876 | 184,465 | NA |
| **No. Contigs** | 125,052 | 234,153 | 114,173 | 259,569 | 265,967 | 260,526 | 10,787,574 |
| **Total Contig Bases (Gb)** | 2.04 | 1.28 | 0.18 | 1.41 | 1.39 | 1.42 | 2.24 |
| **% GC Content** | 46.9 | 46.0 | 45.6 | 46.7 | 46.7 | 46.7 | 46.8 |
| **% Repeat Content** | 76.3 | 65.5 | 11.6 | 69.6 | 69.4 | 69.7 | 75.7 |
| **N50 of Contigs (kb)** | 41.2 | 14.5 | 1.73 | 12.9 | 11.6 | 13.4 | 0.238 |

Table S2. Categorization of trimmed simulation contig alignment.

| **Category** | **No. Contigs Uniquely Aligned to REF (% of total classified)** | | | |
| --- | --- | --- | --- | --- |
|  | **Sim 1^^[[5]](#footnote-5)^^** | **Sim 2^^[[6]](#footnote-6)^^** | **Sim 3^^[[7]](#footnote-7)^^** |  |
| Fully aligned | 240,496  (96.1) | 247,939  (96.3) | 239,648  (95.6) |  |
| With tails | 9,802  (3.91) | 9,502  (3.69) | 10,914  (4.36) |  |
| Unclassified | 5,663 | 5,063 | 6,162 |  |
| Total Classified | 250,298 | 257,441 | 250,562 |  |

1. Reference genome and contigs from B73 AGPv2. [↑](#footnote-ref-1)
2. Simulation 1: 50kb molecule length and 400M reads [↑](#footnote-ref-2)
3. Simulation 2: 80kb molecule length and 400M reads [↑](#footnote-ref-3)
4. Simulation 3: 50kb molecule length and 800M reads [↑](#footnote-ref-4)
5. Simulation 1: 50kb molecule lengths and 400M reads [↑](#footnote-ref-5)
6. Simulation 1: 80kb molecule lengths and 400M reads [↑](#footnote-ref-6)
7. Simulation 1: 50kb molecule lengths and 800M reads [↑](#footnote-ref-7)
